# Wave Propagation Analysis of Edge Cracked Circular Beams under Impact Force

**DOI:** 10.1371/journal.pone.0100496

**Published:** 2014-06-27

**Authors:** Şeref Doğuşcan Akbaş

**Affiliations:** Department of Civil Engineering, Bursa Technical University, Bursa, Turkey; Technion - Israel Institute of Technology, Israel

## Abstract

This paper presents responses of an edge circular cantilever beam under the effect of an impact force. The beam is excited by a transverse triangular force impulse modulated by a harmonic motion. The Kelvin–Voigt model for the material of the beam is used. The cracked beam is modelled as an assembly of two sub-beams connected through a massless elastic rotational spring. The considered problem is investigated within the Bernoulli-Euler beam theory by using energy based finite element method. The system of equations of motion is derived by using Lagrange's equations. The obtained system of linear differential equations is reduced to a linear algebraic equation system and solved in the time domain by using Newmark average acceleration method. In the study, the effects of the location of crack, the depth of the crack, on the characteristics of the reflected waves are investigated in detail. Also, the positions of the cracks are calculated by using reflected waves.

## Introduction

Elastic wave propagation through the monitored part is of considerable interest in many fields. The most striking example of the engineering applications is detection of damage or/and material difference in the investigated media. By investigating the character of waves, the type and position of damage or/and different material can be determined.

Structural elements are subjected to destructive effects in the form of initial defects within the material or caused by fatigue or stress concentration. As a result of destructive effects, cracks occur in the structural elements. It is known that a crack in structure elements introduces a local flexibility, becomes more flexible and its dynamic and static behaviours will be changed. Cracks cause local flexibility and changes in structural stiffness. Therefore, understanding the mechanical behavior and the safe performance of edge-cracked structures are importance in designs.

Beams can be found in many different sizes and shapes in the engineering applications. Circular beams are the most used and preferred in the machine elements because of high energy absorbing capability and high buckling strength. Hence, understanding the mechanical behaviour circular beams are very important.

In the last decades, much more attention has been given to the elastic wave propagation of beam structures. Teh and Huang [1] studied an analytical model, based on the elasticity equations, to investigate wave propagation in generally orthotropic beams. A finite element technique is developed for studying the flexural wave propagation in elastic Timoshenko and Bernoulli-Euler beams by Yokoyama and Kishida [2]. Wave propagation in a split beam is analyzed by treating each section separately as a waveguide and imposing appropriate connectivities at their joints by Farris and Doyle [3]. A direct mathematical approach method is developed to study the problem of coupled longitudinal and flexural wave propagation in a periodically supported infinite long beam by Lee and Yeen [4]. A spectral super-element model was used in Gopalakrishnan and Doyle [5] to model transverse crack in isotropic beam and the dynamic stress intensity factor was obtained accurately under impact type loading. Palacz and Krawczuk [6] investigated longitudinal wave propagation in a cracked rod by using the spectral element method. The use of the wave propagation approach combined with a genetic algorithm and the gradient technique for damage detection in beam-like structure is investigated by Krawczuk [7]. Krawczuk et al. [8] studied a new finite spectral element of a cracked Timoshenko beam for modal and elastic wave propagation analysis. Usuki and Maki [9] formulated an equation of motion for a beam according to higher-order beam theory using Reissner's principle. They derived the Laplace transform of the equation and investigated wave-propagation behavior under transverse impact. A method of crack detection in beam is provided by wavelet analysis of transient flexural wave by Tian et al. [10]. Kang et al. [11] applied the wave approach based on the reflection, transmission and propagation of waves to obtain the natural frequencies of finite curved beams. A spectral finite element with embedded transverse crack is developed and implemented to simulate the diagnostic wave scattering in composite beams with various forms of transverse crack by Kumar et al. [12]. The wave propagation model investigated herein is based on the known fact that material discontinuities affect the propagation of elastic waves in solids by Ostachowicz et al. [13]. A spectral finite element model for analysis of flexural-shear coupled wave propagation in laminated and delaminated, multilayer composite beams is presented by Palacz et al. [14], [15]. A new spectral element is formulated to analyse wave propagation in an anisotropic inhomogeneous beam by Chakraborty and Gopalakrishnan [16]. Watanabe and Sugimoto [17] studied flexural wave propagation in a spatially periodic structure consisting of identical beams of finite length. Vinod et al. [18] investigated a formulation of an approximate spectral element for uniform and tapered rotating Euler–Bernoulli beams. Sridhar et al. [19] investigated the development of an effective numerical tool in the form of pseudospectral method for wave propagation analysis in anisotropic and inhomogeneous structures. An experimental method of detecting damage using the flexural wave propagation characteristics is proposed by Park [20]. Chouvion et al. [21] studied a systematic wave propagation approach for the free vibration analysis of networks consisting of slender, straight and curved beam elements and complete rings. Frikha et al. [22] investigated physical analysis of the effect of axial load on the propagation of elastic waves in helical beams. Kocatürk et al.[23] studied wave propagation of a piecewise homegenous cantilever beam under impact force. Kocatürk and Akbas [24] investigated wave propagation of a microbeam with the modified couple stress theory. In a recent study, wave propagation and localization in periodic and randomly disordered periodic piezoelectric axial-bending coupled beams are studied by Zhu et al. [25]. Akbaş [26] studied the effect of the elastic foundation types on the wave propagation of the beams.

A better understanding of the mechanism of how the crack effects change response of wave propagation of a circular beam is necessary, and is a prerequisite for further exploration and application of the cracked circular beams.

In this study, wave propagation in a cantilever circular beam under the effect of an impact force is studied. The considered problem is investigated within the Bernoulli-Euler beam theory by using energy based finite element method. The Kelvin–Voigt model for the material of the beam is used. The cracked beam is modelled as an assembly of two sub-beams connected through a massless elastic rotational spring. The system of equations of motion is derived by using Lagrange's equations. The obtained system of linear differential equations is reduced to a linear algebraic equation system and solved in the time domain by using Newmark average acceleration method. The effects of the location of crack, the depth of the crack, on the characteristics of the reflected waves are investigated in detail. Also, the positions of the cracks are calculated by using reflected waves.

## Theory and Formulations

**Figure 1:**
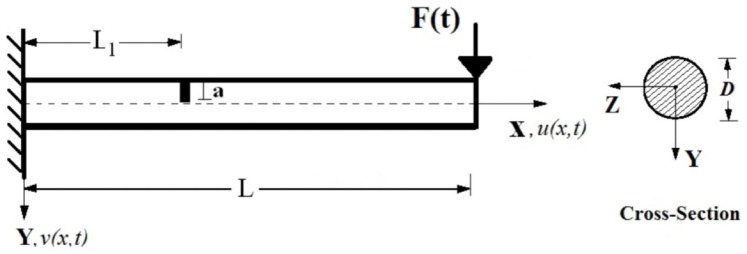
A circular beam with an open edge crack subjected to an impact force.

Consider a beam of length *L*, diameter *D*, containing an edge crack of depth *a* located at a distance 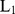 from the left end, as shown in Fig. 1. One of the supports of the beam is assumed to be fixed and the other free. The beam is subjected to an impact force in the transverse direction as seen from Fig. 1. It is assumed that the crack is perpendicular to beam surface and always remains open.

### Governing equations of intact beam

The beam is modeled within the Euler-Bernoulli beam theory. According to the coordinate system *(X,Y,Z)* shown in Fig. 1, based on Euler-Bernoulli beam theory, the axial and the transverse displacement field are expressed as
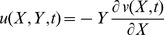
(1)

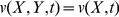
(2)

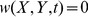
(3)Where *u*,*v* and *w* are *x*,*y* and *z* components of the displacement vector *q*, respectively, and *t* indicates time.

Because the transversal surfaces of the beam is free of stress, then
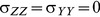
(4)The Kelvin–Voigt model for the material is used. The constitutive relations for the Kelvin–Voigt model between the stresses and strains become
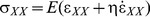
(5)where *E* indicates the Young's modulus of the beam, 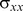 indicates normal stresses, 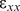 indicates normal strains in the *X* direction, 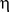 indicates the damping ratios, as follows
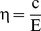
(6)where c indicates the coefficient of damping of the beam. By using Eqs. (1) and (2), the strain- displacement relation can be obtained:
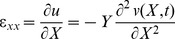
(7)The potential energy of the beam is follows
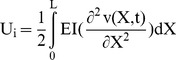
(8)Where *I* is the inertia moment of the beam. The kinetic energy of the beam at any instant *t* is
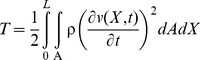
(9)Where 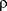 is the mass density of the beam. The potential energy of the external load can be written as
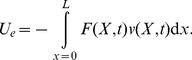
(10)The dissipation function of the beam at any instant *t* is
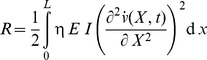
(11)Lagrangian functional of the problem is given as follows:
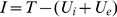
(12)


### Solution method of the problem

**Figure 2:**
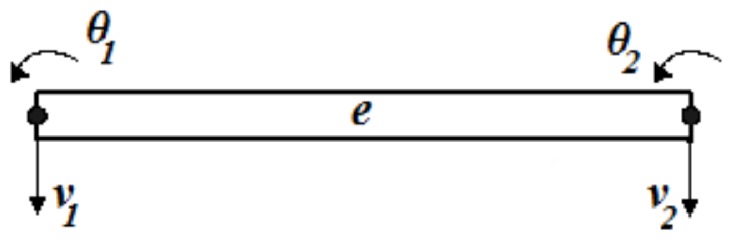
A two-node beam element.

The considered problem is solved by using Lagrange's equations and time integration method of Newmark [27]. In order to apply the Lagrange's equations, the displacements of nodes of the unknown functions *q* (X,t) which is written for a two-node beam element shown in Fig. 2 are defined as follows
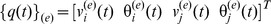
(13)


The displacement field of the finite element is expressed in terms of nodal displacements as follows
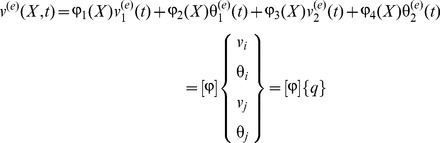
(14)where 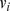 and 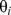 are transverse displacements and slopes at the two end nodes of the beam element, respectively. 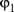, 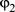, 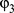 and 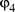 are interpolation functions and given as follows:
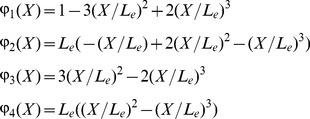
(15)where *L_e_* is the length of the beam element.

By substituting Equations (14) into Equations (8), (9) and (11), energy functions can be rewritten as follows:
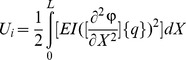
(16)

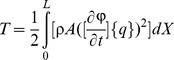
(17)

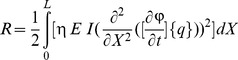
(18)The Lagrange's equations gives the following equation;
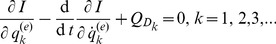
(19)where
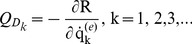
(20)

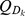
 is the generalized damping load which can be obtained from the dissipation function by differentiating *R* with respect to 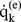.

The Lagrange's equations yield the system of equations of motion for the finite element and by use of usual assemblage procedure the following system of equations of motion for the whole system can be obtained as follows
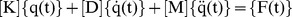
(21)where
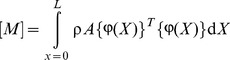
(22)

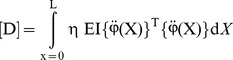
(23)

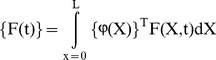
(24)

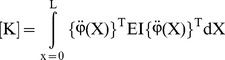
(25)where, 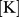 is the stiffness matrix, 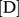 is the damping matrix, 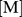 is mass matrix and 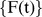 is the load vector. The motion equations which is given by Eq. (21), are solved in the time domain by using Newmark average acceleration method (Newmark [27]).

### Crack modeling

**Figure 3:**
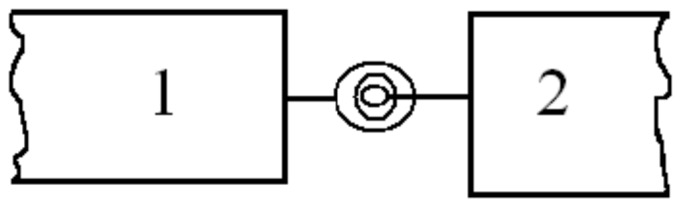
Rotational spring model.

The cracked beam is modeled as an assembly of two sub-beams connected through a massless elastic rotational spring shown in Fig. 3.

**Figure 4:**
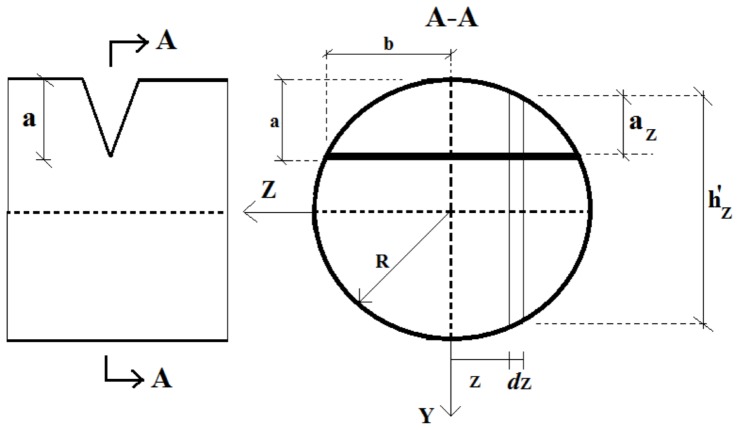
The geometry of the cracked circular cross section.

The bending stiffness of the cracked section 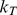 is related to the flexibility G by
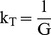
(26)Cracked section's flexibility G can be derived from Broek's approximation (Broek [28]):
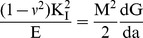
(27)where *M* is the bending moment at the cracked section, 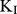 is the stress intensity factor (SIF) under mode I bending load and is a function of the geometry and the loading properties as well. 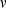 indicates Poisson's ratio. For circular cross section, the stress intensity factor for 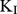 a single edge cracked beam specimen under pure bending *M* can be written as follow (Tada et al. [29])
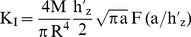
(28)Where
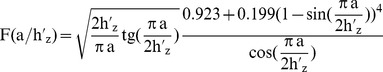
(29)Where *a* is crack of depth and 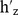 is the height of the strip, is shown Fig. 4, and written as
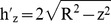
(30)where *R* is the radius of the cross section of the beam.

After substituting Eq. (28) into Eq. (27) and by integrating Eq. (27), the flexibility coefficient of the crack section G is obtained as
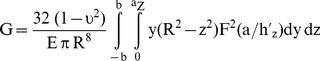
(31)where *b* and 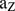 are the boundary of the strip and the local crack depth respectively, are shown in Fig. 4, respectively, and written as
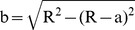
(32)

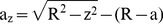
(33)The spring connects the adjacent left and right elements and couples the slopes of the two beam elements at the crack location. In the massless spring model, the compatibility conditions enforce the continuities of the axial displacement, transverse deflection, axial force and bending moment across the crack at the cracked section (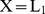), that is,
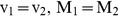
(34)The discontinuity in the slope is as follows:
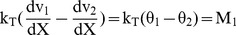
(35)Based on the massless spring model, the stiffness matrix of the cracked section as follows:
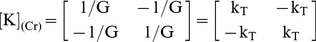
(36)The stiffness matrix of the cracked section is written according to the displacement vector:
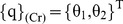
(37)Where 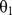 and 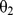 are the angles of the cracked section. With adding crack model, the equations of motion for the finite element and by use of usual assemblage procedure the following system of equations of motion for the whole system can be obtained as follows:
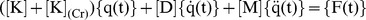
(38)


## Numerical Results

**Figure 5:**
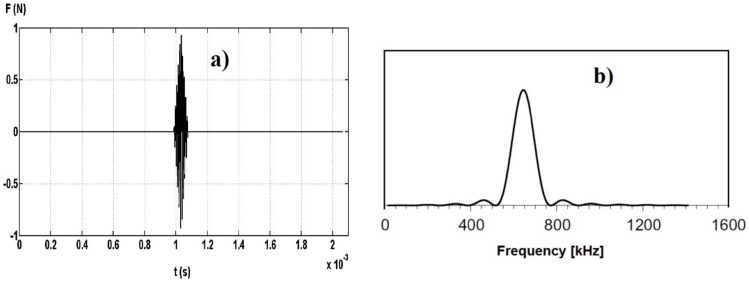
The shape of the excitation impulse in the a) time domain and b) frequency domain [13].

In the numerical examples, the effects of the location of crack, the depth of the crack, on the characteristics of the reflected waves are presented. In the numerical study, the physical properties of the beam are Young's modulus *E* = 70 *GPa*, Poisson's ratio *ν* = 0,3, mass density *ρ* = 2700 *kg/m^3^* and the damping ratio 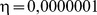. The geometrical properties of the pile are length *L* = 3 *m* and the diameter *D* = 2 cm. The problem is analyzed within the framework of the Bernoulli–Euler beam theory. Numerical calculations in the time domain are made by using Newmark average acceleration method. The system of linear differential equations which are given by Equation (20), is reduced to a linear algebraic system of equations by using average acceleration method. In the numerical calculations, the number of finite elements is taken as *n* = 100. The beam is excited by a transverse triangular force impulse (with a peak value 1 N) modulated by a harmonic function (Fig. 5) (Ostachowicz et al., [13]). In this study, higher frequency excitation impulse is used for detection of the cracks. The frequencies used in this technique are much higher than those typically used in modal analysis based methods but are lower than the frequencies used for ultrasonic testing. In this study, the excited frequencies lies in the range between 200 and 1200 kHz, with dominant one about 700 kHz. At such high frequencies, the response is dominated by the local mode and the wavelength of the excitation is small enough to detect incipient or potentially significant damage. (Ostachowicz et al., [13]). By using the equation of the wave propagation speed, the wave propagation speed is calculated as approximately *v* = 4221,5 m/s.

**Figure 6:**
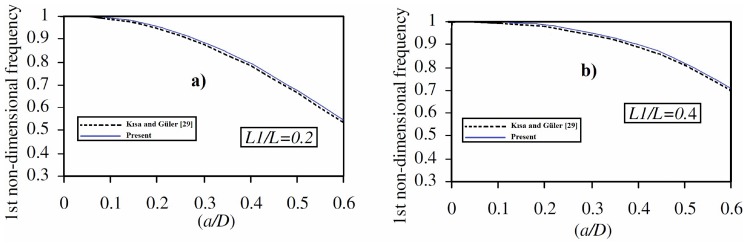
The relationship between first non-dimensional natural frequency and the crack depth ratio for different crack locations. a) *L_1_/L* = 0.2, b) *L_1_/L* = 0.4.

In order to establish the accuracy of the present formulation and the computer program developed by the author, the results obtained from the present study are compared with the available results in the literature. For this purpose, the first fundamental frequency of a cantilever circular beam with an open edge are calculated for different the location of crack ((*L_1_/L*)) and the crack depth ratios for *L* = 2 m, *D* = 0.4 m, *E = 216 GPa*, *ρ* = 7850 *kg/m^3^*, *ν* = 0,33 and compared with those of Kısa and Güler [30] in the Figure 6. As seen from Figure 6, the present results are close to the results of Kısa and Güler [30].

**Figure 7:**
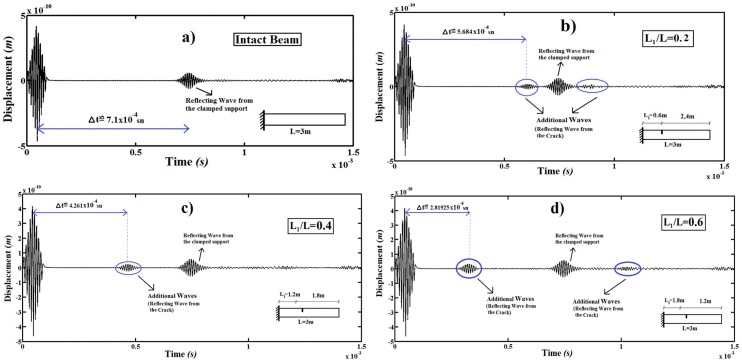
Transverse displacement at the free end of the beam. a) Intact beam, b) *L_1_/L* = 0.1, c) *L_1_/L* = 0.3 and d) *L_1_/L* = 0.5.

Fig. 7 illustrates the transverse displacements at the free end of the cantilever beam for different the crack locations (*L_1_/L*) for the crack depth ratio *a/D* = 0.2.

It is seen from Fig. 7 that the crack location affects significantly the wave propagation of the beam. It is seen from Fig. 6a that two waves occur (namely the excitation and the reflected wave) in the intact case. In Fig. 7a, the second wave occurs because of reflecting from the boundaries of the clamped support. In the case of the crack, additional secondary waves generate with first primary waves (see the circles) in the Fig. 7b, Fig. 7c and Fig. 7d. The additional secondary waves occur because of reflecting from the cracks. Also, it is seen from Fig. 7 that the crack locations get closer to the free end of the beam (namely, with the crack locations ratio (*L_1_/L*) increases), the additional secondary waves appear significantly and the amplitude of additional secondary waves increase considerably. When the crack locations get closer to the clamped support, additional secondary waves interfere with first primary waves. This is because, with decrease in the distance between clamped support and crack, the reflected waves of the clamped support and the crack interfere with each other. The crack locations get closer to the free end, the distance between first primary waves and additional secondary waves increase significantly.

The position of the cracks and supports can be calculated by using wave propagation analysis.

For this purpose, the position of the clamped support and the cracks are calculated by using Fig. 7.

Firstly, the position of the clamped support is calculated by using Fig. 7a: The time interval from the first wave (the excitation wave) and the second wave (reflected wave from the clamped support) is 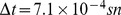
(see Fig. 7a). It should be noted again that the wave propagation speed is *v* =  4221.5 m/s.

By using the relationship between velocity and time, the position of clamped support from the end of the beam can be calculated as follows;
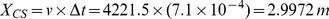
As seen from the result, the present result is very close to the real position of the clamped support from the end of the beam (*L = 3 m*).

The positions of the cracks are calculated by Fig. 7b, Fig. 7c, and Fig. 7d for *L_1_/L = 0.2, L_1_/L = 0.4* and *L_1_/L = 0.6*, respectively.

### 

#### For *L_1_/L = 0.2*

The time interval from the first wave (the excitation wave) and the second wave (additional wave from the crack) is 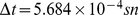
(see Fig. 7b). The position of the crack from the end of the beam is calculated as follows;
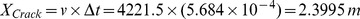
It is seen from the result, the present result is very close to the real position of the crack from the end of the beam *L = 2.4 m* as shown Fig. 7b.

*For L_1_/L = 0.4:*

The time interval from the first wave (the excitation wave) and the second wave (additional wave from the crack) is 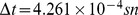
(see Fig. 7c). The position of the crack from the end of the beam is calculated as follows;
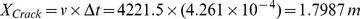
It is seen from the result, the present result is very close to the real position of the crack from the end of the beam *L = 1.8 m* as shown Fig. 7c.

*For L_1_/L = 0.6:*

The time interval from the first wave (the excitation wave) and the second wave (additional wave from the crack) is 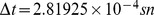
(see Fig. 7d). The position of the crack from the end of the beam is calculated as follows;
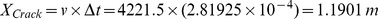
It is seen from the result, the present result is very close to the real position of the crack from the end of the beam *L = 1.2 m* as shown.

It is deduced from Fig. 7 and results that the location of the cracked section can be established by investigating the additional secondary waves.

**Figure 8:**
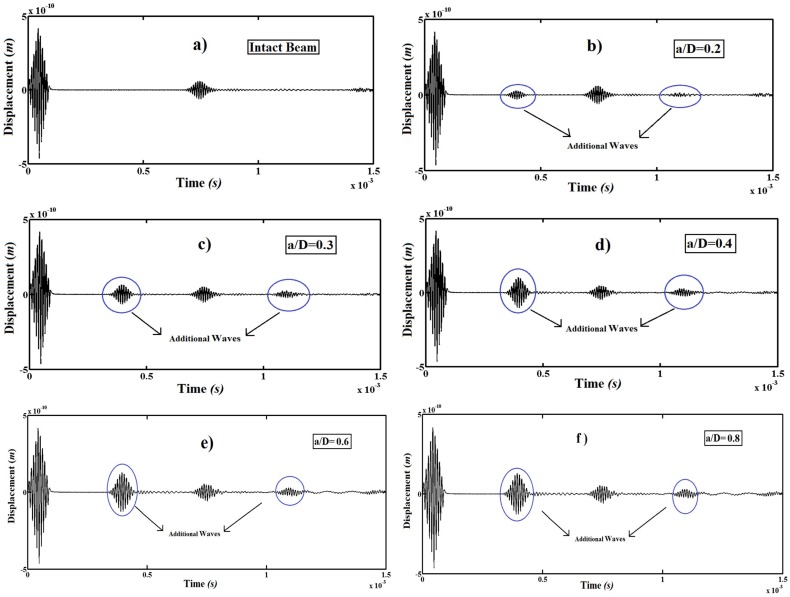
Transverse displacement at the free end of the beam. a) Intact beam, b) *a/D* = 0.2, c) *a/D* = 0.3, d) *a/D* = 0.4, e) *a/D* = 0.6 and f) *a/D* = 0.8.

In Fig. 8, the transverse displacements at the free end of the cantilever beam for different the crack depth ratios (*a/D*) for the crack location *L_1_/L* = 0.5.

As seen from Fig. 8, with the crack depth increase, the amplitude of additional wave increases considerably. This is because by increasing in the crack depth, the strength of the material decreases. Hence, the beam becomes more flexible. Also, it is seen from Fig. 8 that the generation time and location of the additional waves are same for different the crack depth ratios (*a/D*). This is because; the location of the crack is not changed. It shows that by using wave propagation analysis, structural damages can be detected easily.

**Figure 9:**
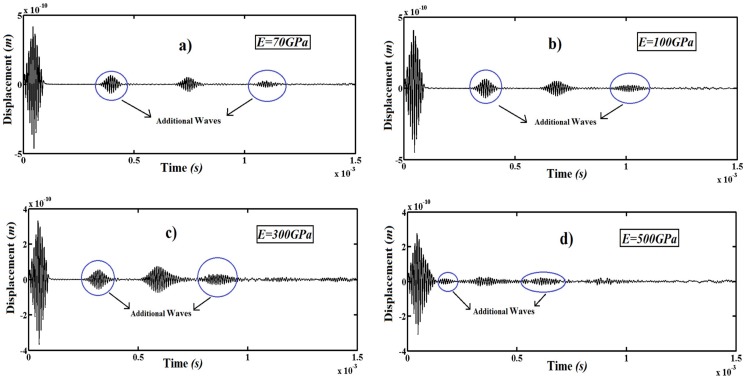
Transverse displacement at the free end of the beam. a) *E* = 70 *GPa*, b) *E* = 100 *GPa*, c) *E* = 300 *GPa* and d) *E* = 500 *GPa*.

In Fig. 9, the effect of the Young's modulus *E* on the wave propagation of the beam is shown for *a/D* = 0.3 and *L_1_/L* = 0.5.

It is seen from Fig. 9 that Young's Modulus plays an important role in the additional waves. With the increased Young's Modulus *E*, the amplitude of the additional wave dramatically decreases. This is because by increasing in Young's Modulus, the strength of the material increases. Also, it is observed another result of Fig. 9 that with the increased Young's Modulus *E*, the generation time and location of the primary and additional waves decreases. It is deduced from Fig. 9 that Young's Modulus is very effective for reducing the negative influence of the cracks.

## Conclusions

Wave propagation in an edge circular cantilever beam under the effect of an impact force is investigated. The effects of the location of crack, the depth of the crack and Young's Modulus on the wave propagations of the circular beam are investigated in detail. The following conclusions are reached from the obtained results:

1. The wave propagation analysis can easily be used for crack detection procedures within structures.
2. The crack locations can be established by investigating in the additional waves.
3. The magnitude of the crack is very effective in the amplitude of additional waves.
4. The crack locations and the crack depth have a great influence on the wave propagation of the circular beam.
5. There are significant differences of the wave propagation for the cracked and intact circular beams.
6. Young's Modulus is very effective for reducing the negative influence of the cracks.
